# High activity CAZyme cassette for improving biomass degradation in thermophiles

**DOI:** 10.1186/s13068-018-1014-2

**Published:** 2018-02-01

**Authors:** Roman Brunecky, Daehwan Chung, Nicholas S. Sarai, Neal Hengge, Jordan F. Russell, Jenna Young, Ashutosh Mittal, Patthra Pason, Todd Vander Wall, William Michener, Todd Shollenberger, Janet Westpheling, Michael E. Himmel, Yannick J. Bomble

**Affiliations:** 1 Biosciences Center, National Renewable Energy Laboratory, 15013 Denver West Parkway, Golden, CO 80401 USA; 2 Department of Genetics, University of Georgia, Athens, GA 30602 USA; 3 Development and Training Institute, King Mongkut’s University of Technology Thonburi, Bangkok, Thailand

**Keywords:** Biofuels, Biomass degrading enzymes, Cellulose, Biomass, Thermophile, *Caldicellulosiruptor bescii*, Anaerobe

## Abstract

**Background:**

Thermophilic microorganisms and their enzymes offer several advantages for industrial application over their mesophilic counterparts. For example, a hyperthermophilic anaerobe, *Caldicellulosiruptor bescii*, was recently isolated from hot springs in Kamchatka, Siberia, and shown to have very high cellulolytic activity. Additionally, it is one of a few microorganisms being considered as viable candidates for consolidated bioprocessing applications. Moreover, *C. bescii* is capable of deconstructing plant biomass without enzymatic or chemical pretreatment. This ability is accomplished by the production and secretion of free, multi-modular and multi-functional enzymes, one of which, *Cb*Cel9A/Cel48A also known as CelA, is able to outperform enzymes found in commercial enzyme preparations. Furthermore, the complete *C. bescii* exoproteome is extremely thermostable and highly active at elevated temperatures, unlike commercial fungal cellulases. Therefore, understanding the functional diversity of enzymes in the *C. bescii* exoproteome and how inter-molecular synergy between them confers *C. bescii* with its high cellulolytic activity is an important endeavor to enable the production of more efficient biomass degrading enzyme formulations and in turn, better cellulolytic industrial microorganisms.

**Results:**

To advance the understanding of the *C. bescii* exoproteome we have expressed, purified, and tested four of the primary enzymes found in the exoproteome and we have found that the combination of three or four of the most highly expressed enzymes exhibit synergistic activity. We also demonstrated that discrete combinations of these enzymes mimic and even  improve upon the activity of the whole *C. bescii* exoproteome, even though some of the enzymes lack significant activity on their own.

**Conclusions:**

We have demonstrated that it is possible to replicate the cellulolytic activity of the native *C. bescii* exoproteome utilizing a minimal gene set, and that these minimal gene sets are more active than the whole exoproteome. In the future, this may lead to more simplified and efficient cellulolytic enzyme preparations or yield improvements when these enzymes are expressed in microorganisms engineered for consolidated bioprocessing.

**Electronic supplementary material:**

The online version of this article (10.1186/s13068-018-1014-2) contains supplementary material, which is available to authorized users.

## Background

*Caldicellulosiruptor bescii,* the most thermophilic cellulolytic  bacterium yet discovered, exhibits high cellulolytic activity on a variety of biomass substrates. It is able to grow on and deconstruct biomass without conventional pretreatments [1, 2]. The carbohydrate-active enzymes (CAZymes) that *C. bescii* uses for biomass deconstruction are different from those used by other bacteria and fungi [3]. Nature has evolved cellulolytic microbes that produce a wide variety of enzymes that act on plant biomass using diverse deconstruction mechanisms. For example, *Clostridium thermocellum* relies on cellulosomes, which are complex protein assemblies attached to the microbial cell to mediate solubilization of plant biomass [4, 5]. However, this deconstruction mechanism is rare and only found in a few anaerobic bacteria and fungi. Most fungi and cellulolytic bacteria produce free enzymes that act synergistically [6, 7]. This system represents the most common deconstruction mechanism for plant biomass in the biosphere [8]. On the other hand, *C. bescii*, like many other *Caldicellulosiruptor* species, relies primarily on a combination of CAZymes comprised of several catalytic domains instead of the canonical single catalytic domain architecture employed by other cellulolytic microorganisms. The *C. bescii* genome contains 88 CAZyme genes with varying degrees of complexity in their architectures, but the most abundant CAZymes in its exoproteome are multi-modular and multi-functional [9]. Cellulolytic ability correlates with the presence of CelA (*Cb*Cel9A/Cel48A) the most abundant CAZyme in the *C. bescii* exoproteome. CelA consists of a Family 9A-CBM3_c_ processive endoglucanase, a Family 48 exoglucanase, and two Family 3b carbohydrate-binding modules (CBM3_b_) connected by proline/threonine rich linker regions [10, 11]. *Cb*Cel9A/Cel48A is so far the most efficient single gene product ever tested for biomass deconstruction and has been shown to be vital for *C. bescii* growth on plant biomass [12]. *Cb*Cel9A/Cel48A represents nearly 50% of the CAZymes in the exoproteome [13], yet there remains a significant fraction of other multi-modular and multi-functional CAZymes. It is becoming clear that the high cellulolytic activity displayed by this system is the result of inter-molecular synergy between these CAZymes [14].

The next three most abundant CAZymes in the exoproteome are *Cb*Cel9B/Man5A, *Cb*Xyn10A/Cel48B, and *Cb*Man5B/Cel44A, respectively. *Cb*Cel9B/Man5A has an N-terminus GH9 catalytic domain linked to a CBM3_c_ module, a construct similar to that of the N-terminus of *Cb*Cel9A/Cel48A, which is also likely to be active on cellulose, as well as two CBM3_b_ modules and a GH5 domain. *Cb*Cel9B/Man5A was previously described as a mannanase [15]. *Cb*Xyn10A/Cel48B contains two catalytic domains, a GH10 and a GH48; as well as two CBM3_b_ modules suggesting it is a xylanase and a processive cellulase. Finally, *Cb*Man5B/Cel44A is comprised of a GH5, a GH44, and two CBM3_b_ modules, which suggests that it is a bifunctional mannanase/endoglucanase or possibly a xyloglucanase. Understanding the synergy between these enzymes during biomass deconstruction is vital to achieving our goal of enhanced understanding of biomass deconstruction in the biosphere including how microorganisms have evolved different sets of CAZymes to efficiently degrade biomass. Furthermore, a deeper understanding is also important to enable the development of thermophilic CAZyme cassettes which may be used in large scale biomass saccharification applications, such as biofuels production. Another nascent application of such cassettes of cell wall degrading enzymes will enable promising non-cellulolytic thermophiles, already producing high titers of biochemical products, to deconstruct biomass within a consolidated bioprocessing (CBP) strategy. Indeed, biomass deconstruction at elevated temperatures presents several advantages in simultaneous saccharification and fermentation (SSF) as well as CBP applications. Thermophilic conditions can allow better enzyme penetration and cell-wall disorganization of the biomass, thermostable enzymes are often more resistant to the relatively harsh conditions of industrial processes with high solvent or high salt concentrations and offer potentially faster reaction kinetics [16–18]. Perhaps most importantly, high temperatures in a fermentative process prevent costly microbial contamination [16–18]. In this study, we show that these thermostable enzymes from *C. bescii* possess strong synergy and that the performance of certain combinations of these enzymes is competitive with that of the entire exoproteome. We suggest that a CAZyme cassette including three or four of these enzymes is enough to recapitulate and even exceed the activity of the *C. bescii* exoproteome, showing they are ideal candidates to confer the ability to degrade cellulosic substrates effectively to non-cellulolytic thermophiles.

## Results and discussions

### Homologous expression and the purification of *Cb*Cel9A/Cel48A, *Cb*Cel9B/Man5A, *Cb*Xyn10A/Cel48B, and *Cb*Man5B/Cel44A

**Fig. 1:**
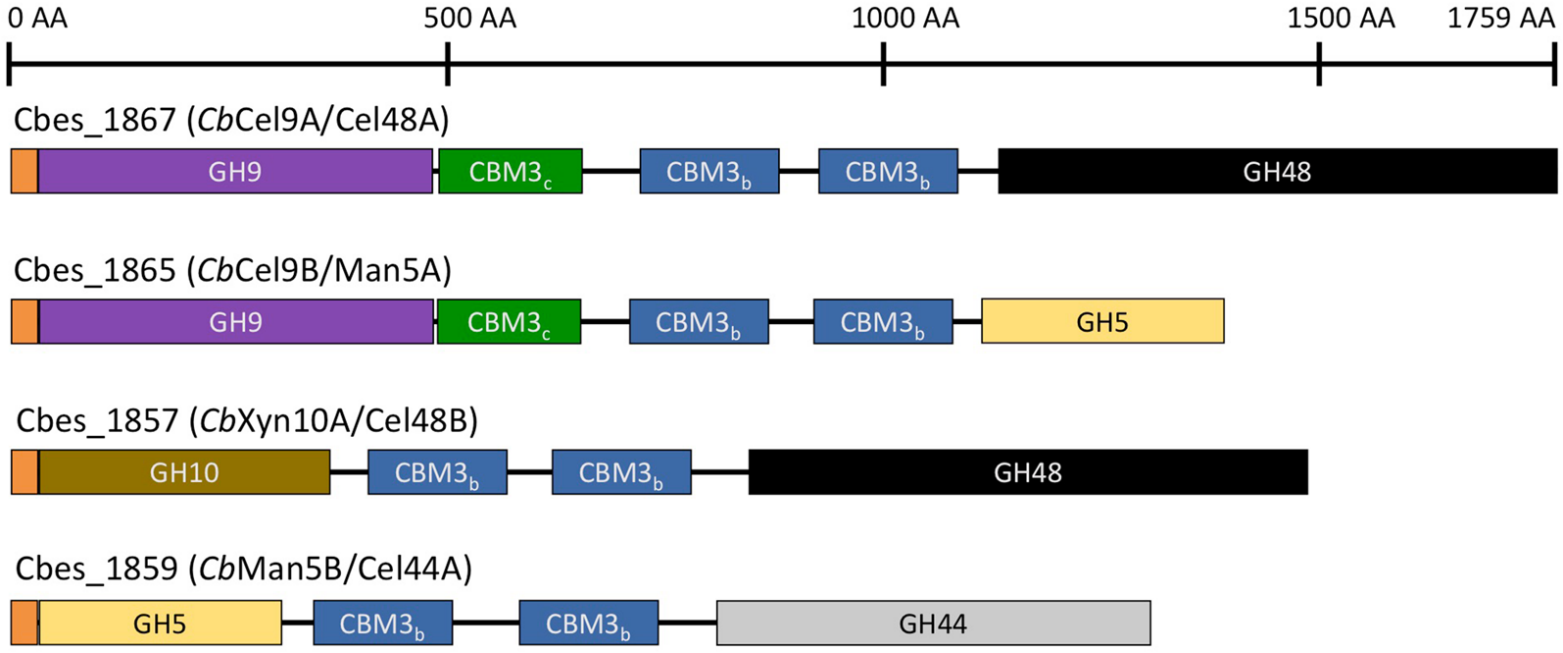
Domain organization of multifunctional CAZymes from the exoproteome of *C. bescii*. CelA, the dominant CAZyme in the exoproteome of *C. bescii* and three additional multifunctional enzymes were homologously expressed. Each enzyme contains two catalytic domains and two or three family 3 carbohydrate binding modules separated by proline/threonine rich linker peptides

To investigate the synergy between four extracellular CAZymes from *C. bescii*, we homologously expressed and purified *Cb*Cel9A/Cel48A (Cbes_1867), *Cb*Cel9B/Man5A (Cbes_1865), *Cb*Xyn10A/Cel48B (Cbes_1857), and *Cb*Man5B/Cel44A (Cbes_1859) (Fig. 1). Expression of these thermophilic extracellular proteins in their native host with correct post-translational modification, in this case glycosylation, is essential to retain native activity and stability, especially at the elevated optimal growth temperature of *C. bescii*. Extensive studies have been performed to biochemically characterize these four proteins, but only *Cb*Cel9A/Cel48A has been purified from its native host [10]. Previous studies for the other three most abundant CAZymes in the exoproteome, *Cb*Cel9B/Man5A, *Cb*Xyn10A/Cel48B, and *Cb*Man5B/Cel44A, utilized proteins expressed in *E. coli* at mesophilic temperature, thus potentially lacking proper post-translational modifications [15, 19–22]. Additionally, all but one of these studies focused only on a catalytic domain from these CAZymes.

**Fig. 2:**
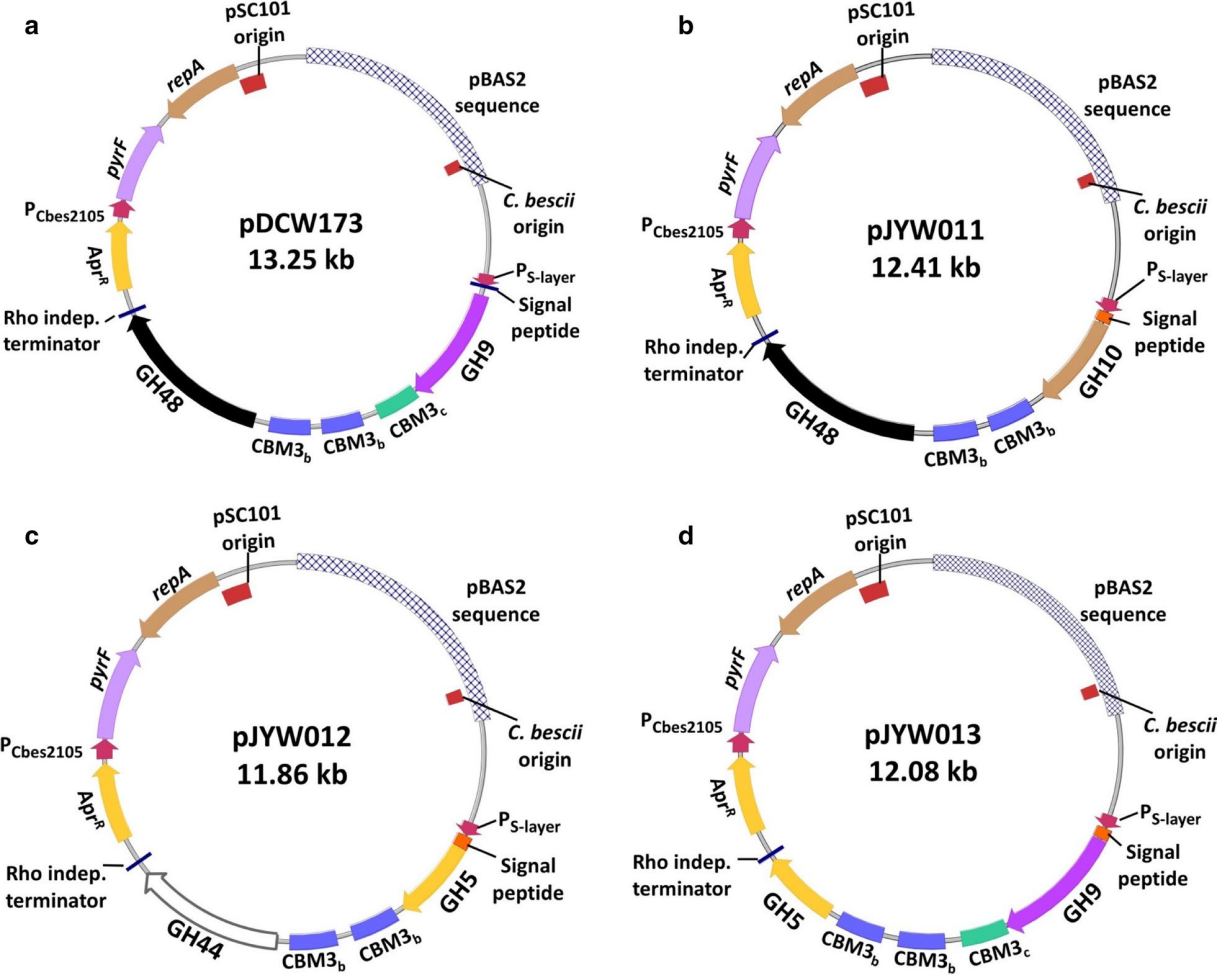
Plasmid maps of *Caldicellulosiruptor bescii* expression vector for **a** Cbes_1867 (WT; GH9-CBM3_c_-CBM3_b_-CBM3_b_-GH48), **b** Cbes_1857 (GH10-CBM3_b_-CBM3_b_-GH48), **c** Cbes_1859 (GH5-CBM3_b_-CBM3_b_-GH44), and **d** Cbes_1865 (GH9-CBM3_c_-CBM3_b_-CBM3_b_-GH5) expression. Various multi-domain cellulases derived from *C. bescii* were expressed under the control of the regulatory region of the *C. bescii* S-layer protein. The expression vectors contain a signal peptide sequence, a C-terminal 6X His-tag, a Rho independent terminator, the *pyrF* (from *C. thermocellum*) cassette for selection, and pBAS2 sequences for replication in *C. bescii.* The apramycin resistant gene cassette (*Apr*^*R*^), pSC101 low copy replication origin in *E. coli*, and *repA*, a plasmid-encoded gene required for pSC101 replication are indicated

To address these issues, we constructed three new expression vectors (Fig. 2) and *C. bescii* expression strains (Additional file 1: Table S1). Diagrams of the domain architecture of four CAZymes employed in this study are shown in Fig. 1, with *Cb*Cel9A/Cel48A being from Chung et al. [23]. These polypeptides contain predicted signal peptides, catalytic domains, and CBMs. Expression vectors were constructed by inserting the gene encoding Cbes_1857, Cbes_1859, and Cbes_1865 into pDCW173, resulting in pJYW011, pJYW012, and pJYW013, respectively (Fig. 2). pDCW173 contains an expression cassette for *Cb*Cel9A/Cel48A and was successfully used to homologously express CelA [10, 23]. The *Cb*Cel9A/Cel48A expression cassette directs transcription of *Cb*Cel9A/Cel48A using the regulatory and rho independent terminator sequences surrounding Cbes_2303 (S-layer protein) and contains a C-terminal 6X histidine-tag (Fig. 1a), followed by a stop codon. We replaced the Cbes_1867 gene by other multifunctional coding genes to create pJYW011, pJYW012, and pJYW013. Plasmid DNA was transformed into JWCB029 (*ΔpyrFA Δldh::ISCbe4 Δcbe1 ΔcelA*) [12] and transformants were selected for uracil prototrophy. The presence of the plasmid in transformants was confirmed by PCR amplification using extracted total DNA as template. Primers (DC228 and DC569) were used to amplify the portion of the plasmid containing the open reading frame of Cbes_1867, Cbes_1857, Cbes_1859, and Cbes_1865. PCR amplified products confirmed the presence of each CAZyme encoding gene within its plasmid (data not shown). The structural stability and maintenance of plasmids in expression strains were assessed by back-transformation of total DNA isolated from four independent *C. bescii* transformants into *E. coli* as described in Chung et al. [27] (data not shown). The SDS-PAGE result in Additional file 1: Figure S1 clearly shows that all protein constructs were successfully expressed, secreted, and purified to homogeneity. We did not detect any significant proteolytic cleavage products or prematurely terminated products, which often occur during expression and purification from *E. coli* (data not shown). The predicted sizes of the proteins, Cbes_1867 (~ 195 kDa), Cbes_1857 (~ 165 kDa), Cbes_1859 (~ 142 kDa), and Cbes_1865 ( ~ 151 kDa) were in accordance with these SDS-PAGE results.

### Substrate specificity and product profiles of *Cb*Cel9A/Cel48A, *Cb*Cel9B/Man5A, *Cb*Xyn10A/Cel48B, and *Cb*Man5B/Cel44A

**Fig. 3:**
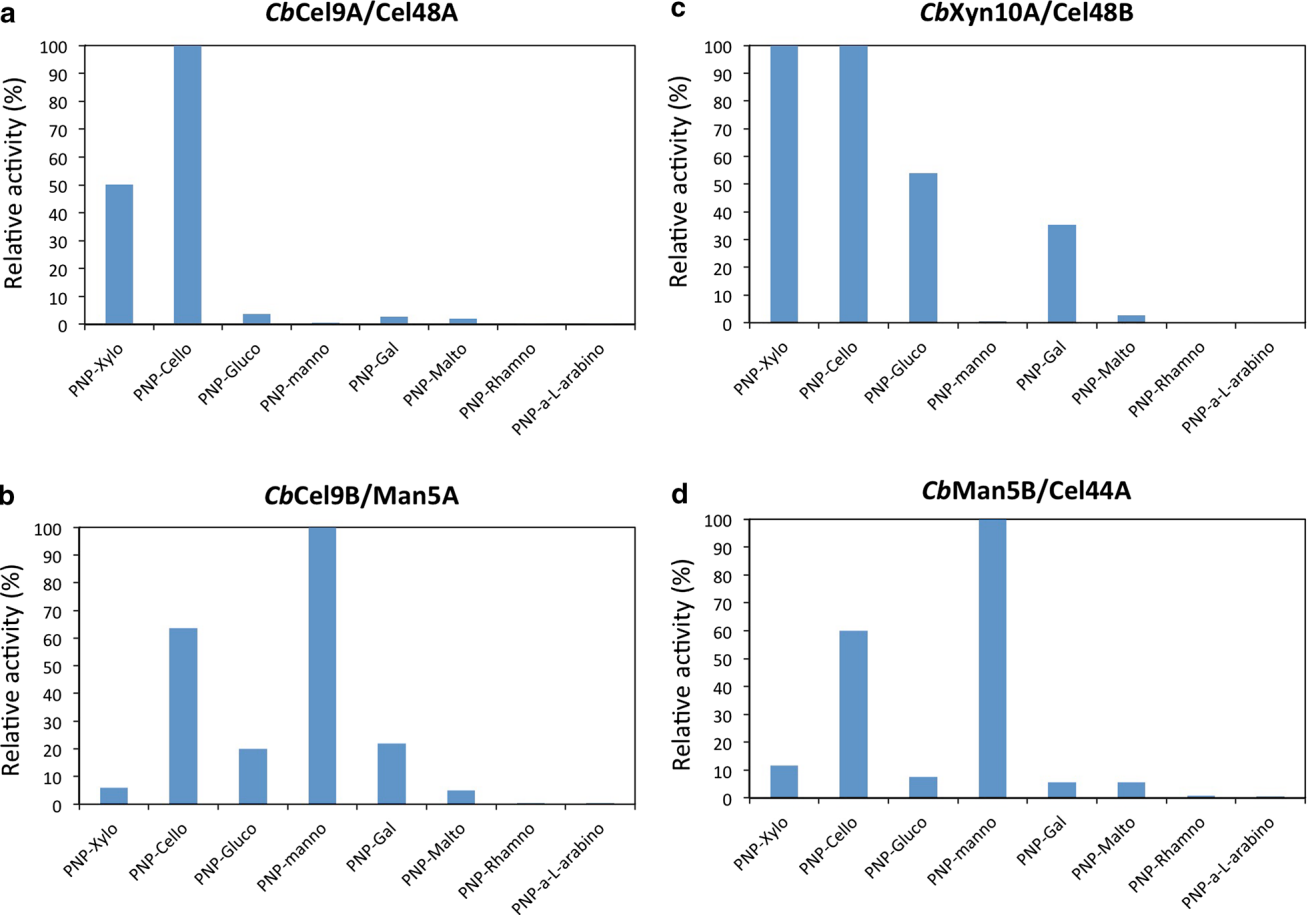
Substrate specificity of *Cb*Cel9A/Cel48A, *Cb*Cel9B/Man5A, *Cb*Xyn10A/Cel48B, and *Cb*Man5B/Cel44A. Relative activity with respect to optimal activity of, **a** *Cb*Cel9A/Cel48A, **b** *Cb*Cel9B/Man5A, **c** *Cb*Xyn10A/Cel48B, **d** *Cb*Man5B/Cel44A, on various PNP substrates as noted

Although we have already characterized *Cb*Cel9A/Cel48A extensively in other publications [10, 11], additional substrate specificity data for this enzyme is included here for completeness. Based on biochemical assays using *p*-nitrophenyl (*p*NP) and azurine cross-linked-labeled (AZCL) derivatives, we identified *Cb*Xyn10A/Cel48B as possessing primarily xylanase activity with some minimal cellulase activity most likely conferred by the GH48 module. One should note that the GH48 of *Cb*Cel9A/Cel48A was also shown to exhibit strong xylanase activity [10]. Activity on galactose and glucose are also possible side activities (Fig. 3; Additional file 1: Figure S2). *Cb*Cel9A/Man5A is primarily identified as a mannanase based on the high activity on *p*NP-mannose and AZCL-mannan, with residual activity on cellulose and xylan. Finally, *Cb*Man5B/Cel44A is also primarily classified as a mannanase, due to its activity on *p*NP-mannan and AZCL-mannan, along with secondary activities as a xylanase and cellulase. The Cel44A module is likely a xyloglucanase as indicated by the AZCL xyloglucanase activity detected.

Single product activity profiles were generated after digesting alkaline peroxide pretreated corn stover (APCS) [11]. The primary products for *Cb*Cel9A/Cel48A were glucose and cellobiose in a 1:2.5 ratio with a trace of cellotriose also detected (Additional file 1: Figure S3). For the xylanase, *Cb*Xyn10A/Cel48B, the primary cello oligomer detected was cellobiose, with very little glucose and cellotriose (Additional file 1: Figure S3). For *Cb*Cel9B/Man5A only glucose and cellobiose were detected in a 1:2.2 ratio (Additional file 1: Figure S3). *Cb*Man5B/Cel44A has low overall activity on glucan, with cellobiose being the primary product, but with both glucose and cellotriose appearing in an approximate 2:1 ratio relative to cellobiose. There was also a trace of cellotetraose detected for *Cb*Man5B/Cel44A in a 6:1 cellobiose/cellotetraose ratio (Additional file 1: Figure S3). The enzymes were also analyzed for xylan release profiles, however, no attempt was made to quantify release. All enzymes, except *Cb*Cel9A/Cel48A, produced xylooligomers up to DP7, whereas *Cb*Cel9A/Cel48A produces xylooligomers up to DP6 (Additional file 1: Table S3).

### Effect of pH and temperature on the activity of *Cb*Cel9A/Cel48A, *Cb*Cel9B/Man5A, *Cb*Xyn10A/Cel48B, and *Cb*Man5B/Cel44A

**Fig. 4:**
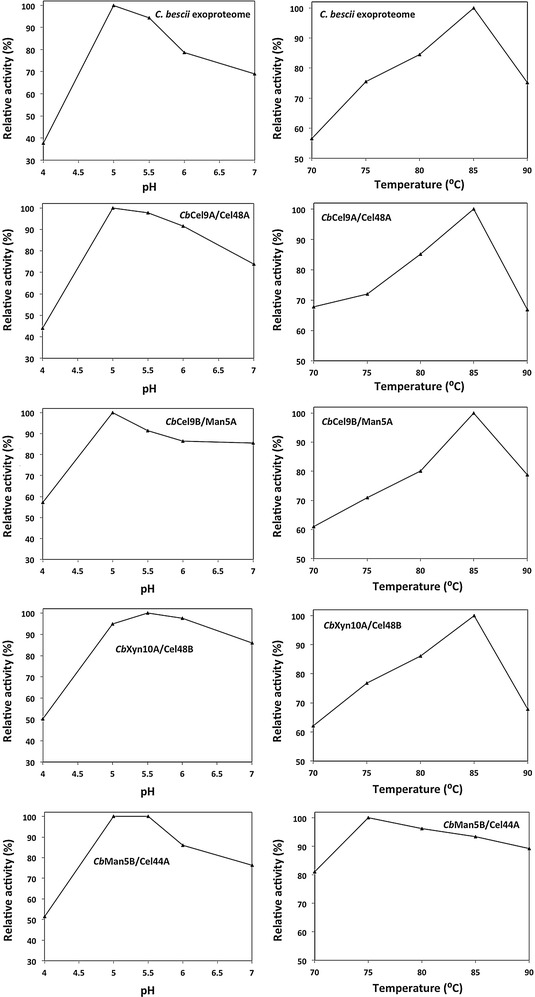
Effect of pH and temperature on the activity of *Cb*Cel9A/Cel48A, *Cb*Cel9B/Man5A, *Cb*Xyn10A/Cel48B, and *Cb*Man5B/Cel44A on APCS. Relative activity remaining with respect to optimal activity for the four CAZymes and the *C. bescii* exoproteome at different pH (4.0–7.0) and temperatures (70–90 °C) on the substrate APCS

Biomass digestion experiments with the single enzymes and the *C. bescii* exoproteome over a range of temperatures (70–90 °C) and pH (4.0–7.0) were carried out on APCS to determine relative activity within these pH and temperature ranges. The optimal temperature for enzyme performance was found to be 85 °C for the *C. bescii* exoproteome, *Cb*Cel9A/Cel48A, *Cb*Xyn10A/Cel48B, and *Cb*Cel9B/Man5A while *Cb*Man5B/Cel44A showed a lower optimal temperature in the range of (75–80 °C) (Fig. 4). These results are similar to previous reports for *Cb*Cel9A/Cel48A on Avicel [10]. Even at elevated temperatures e.g., 90 °C, the single enzymes as well as the *C. bescii* exoproteome showed relative activity greater than 60%. All single enzymes and the *C. bescii* exoproteome appear to have the highest activity in the pH range of 5–5.5 (Fig. 4), within the range of pH reported to be optimal for *C. bescii* CAZymes, *Cb*Cel9A/Cel48A and *C. bescii* exoproteome being commonly used at pH 5.5 [10, 11, 23–25].

### Enzyme performance on pretreated biomass

**Fig. 5:**
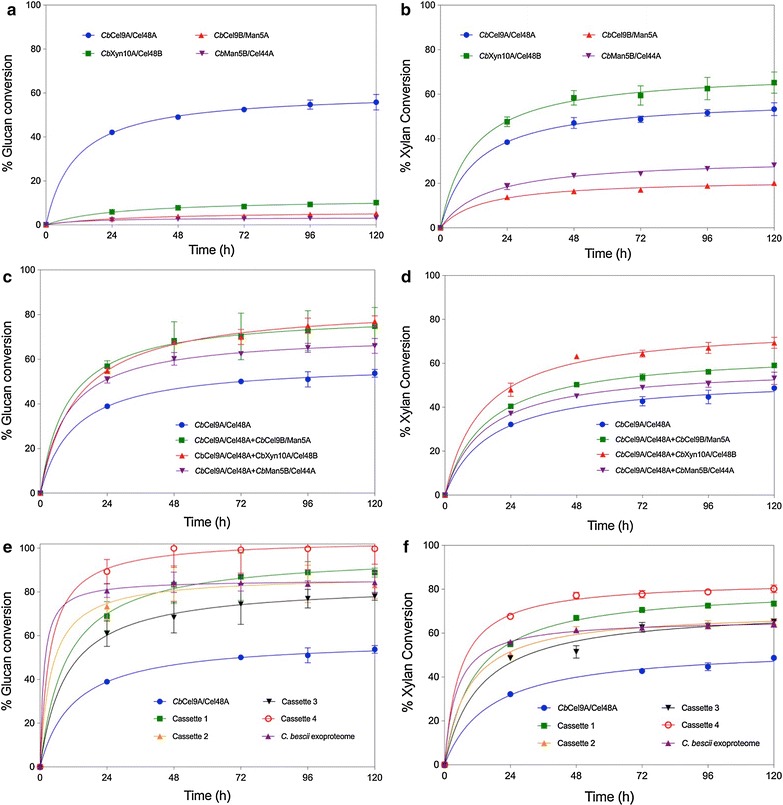
APCS conversion by *Cb*Cel9A/Cel48A, *Cb*Cel9B/Man5A, *Cb*Xyn10A/Cel48B, and *Cb*Man5B/Cel44A. **a** Glucan conversion of APCS by individual *C. bescii* enzymes, **b** xylan conversion of APCS by individual *C. bescii* enzymes, **c** glucan conversion of APCS by binary combinations of *C. bescii* enzymes, **d** xylan conversion of APCS by binary combinations of *C. bescii* enzymes, **e** glucan conversion of APCS by ternary and quaternary combinations of *C. bescii* enzymes, **f** Xylan conversion of APCS by ternary and quaternary combinations of *C. bescii* enzymes. Cassette 1 is a combination of *Cb*Cel9A/Cel48A, *Cb*Cel9B/Man5A, *Cb*Xyn10A/Cel48B, cassette 2 is a combination of *Cb*Cel9A/Cel48A, *Cb*Cel9B/Man5A, *Cb*Man5B/Cel44A, cassette 3 is a combination of *Cb*Cel9A/Cel48A, *Cb*Xyn10A/Cel48B, *Cb*Man5B/Cel44A, cassette 4 is a combination of *Cb*Cel9A/Cel48A, *Cb*Cel9B/Man5A, *Cb*Xyn10A/Cel48B, *Cb*Man5B/Cel44A. All enzyme loadings can be found in the methods section and supplementary information (Additional file 1: Tables S4, S5)

Single enzyme performance was evaluated at an equal enzyme loading using APCS. *Cb*Cel9A/Cel48A, as shown in previous report [10, 11], exhibits high activity on glucan and is the most active enzyme of the set on this biomass substrate component (Fig. 5a). The other enzymes are not as active on glucan with barely 10% conversion compared to close to 60% for *Cb*Cel9A/Cel48A. *Cb*Cel9A/Cel48A also showed the ability to efficiently deconstruct xylan and was the second most active enzyme on this component, with *Cb*Xyn10A/Cel48B being clearly and logically, given its makeup, the best performer on xylan (Fig. 5b). We observed more xylan conversion with *Cb*Cel9B/Man5A and *Cb*Man5B/Cel44A than expected from the preliminary *p*NP and AZCL assays. This may be due to the fact that the synthetic pNP substrates may not always fit well into the active sites of enzymes and is a known limitation of the pNP assay in general. Moreover, single enzyme analysis suggests that *Cb*Cel9A/Cel48A is the major enzyme in the *C. bescii* exoproteome. Given the low glucan conversion rate demonstrated by *Cb*Cel9B/Man5A, we conclude that this enzyme acts on glucan similar to a traditional endo pair with low specific rates when compared to processive enzymes. *Cb*Xyn10A/Cel48B appears to be the major xylan degrading enzyme in the *C. bescii* exoproteome based on the fact that it converts xylan at a slightly higher rate than *Cb*Cel9A/Cel48A (65 and 55%, respectively). *Cb*Man5B/Cel44A is likely to be a bi-functional non-processive endo type of enzyme. Interestingly all of the *C. bescii* CAZymes have activity on xylan even though they are not in all cases comprised of glycoside hydrolases with obvious xylanase activity.

### Synergism between *Cb*Cel9A/Cel48A and multiple CAZymes, comparison to the exoproteome: the making of an efficient and tractable CAZyme cassette

We developed these digestion experiments and selected enzyme loadings using the ratios in which these CAZymes occur in the *C. bescii* exoproteome (Additional file 1: Tables S4, S5). As stated above, *Cb*Cel9A/Cel48A is the major biomass active enzyme present in the exoproteome; when combined with either *Cb*Cel9B/Man5A or *Cb*Xyn10A/Cel48B we observe a synergistic increase in activity from about 50–80% in glucan conversion on APCS (Fig. 5c). Again, these digestion experiments were conducted at the same enzyme ratios as in the exoproteome [13]. We observed that when multiple enzymes are added, rapid hydrolysis of the substrate occurs (see Fig. 5e) and we could thus recapitulate the extent of digestion achieved by the whole exoproteome with the addition to *Cb*Cel9A/Cel48A of *Cb*Cel9B/Man5A and *Cb*Xyn10A/Cel48B or *Cb*Cel9B/Man5A and *Cb*Man5B/Cel44A. Ultimately if all four CAZymes are used together (including *Cb*Cel9A/Cel48A) this quaternary CAZyme cassette can rapidly convert all of the substrate present to an even greater extent than the native exoproteome (Fig. 5e). This is likely due to a higher loading of active components when compared to the whole exoproteome, which likely contains other non-biomass active enzymes such as proteases and other enzymes even though we know that these are in low abundance from proteomic analyses [13]. This quaternary CAZyme cassette could also be exploited to deal with a wide variety of natural substrates by modifying the ratio of each CAZyme present. In our experiments we attempted to replicate the natural ratios of enzymes present in the whole *C. bescii* exproteome when grown on a crystalline substrate as described by Lochner et al., however, when dealing with different natural substrates the composition of the exproteome is likely different [13]. Nevertheless, we would expect similar improvements could be realized on native substrates by altering enzyme ratios.

Xylan conversion follows a somewhat similar pattern to that found for glucan conversion—addition of any single enzyme improves the overall extent of conversion when compared to CbCel9A/Cel48A alone. The best addition, unsurprisingly, utilizes the *Cb*Xyn10A/Cel48B xylanase (Fig. 5d). However, given that this enzyme is even more active on xylan than CbCel9A/Cel48A this effect is not as pronounced. Addition of multiple enzymes again results in the rapid conversion of xylan and addition of any two components equals the performance of the whole exoproteome (Fig. 5f). Similar to the glucan conversion results, cassette 1 with three enzymes or cassette 4 with all four enzymes show dramatically improved performance when compared to the exoproteome (Fig. 5f). It should be noted that while the conversion of glucan by cassette 4 reached 100%, the conversion of xylan reached 80% at the 120 h time point, but given that the whole exoproteome reached only 60% conversion in the same amount of time a likely explanation is that end product inhibition is occurring for the xylan degrading enzymes as there is a significant amount of xylobiose present at the end of the digestion and the addition of a thermostable β-xylosidase would likely further improve the performance of cassette 4. Furthermore, when considering the CAZyme cassette and the natural exoproteome in the broader context of life in a hot springs the uptake of sugars by the organism itself and rapid diffusion at high temperatures would keep end product inhibition to a minimum. Alternatively, in nature, there may also be other thermophilic enzymes present in a hot springs pool that may have thermostable β-xylosidase enzymes that would further enhance the degradation of plant biomass.

These findings, when combined with our previous work demonstrating that the addition of the endoglucanase *Ac*Cel5A from *Acidothermus cellulolyticus* dramatically improves the activity of the whole exoproteome, makes our outlook to engineer more active CAZyme cassettes very promising [24].

## Conclusions

The *C. bescii* proteome is a rich source of highly active hyperthermophilic enzymes. Individually, *Cb*Cel9A/Cel48A is by far the most active enzyme in the exoproteome on glucan and the second most active on xylan. The next most abundantly expressed enzymes have marginal activity on glucan but significant activity on xylan and mannan. Interestingly, these enzymes show high degrees of synergy in the deconstruction of glucan from biomass. This natural condition of *C. bescii* may signal the reality of life in hot springs, where the likelihood of an array of other lignocellulolytic species to reduce the burden of enzyme production is diminished.

Indeed, by tailoring an enzyme cassette with four enzymes we were able to achieve a higher extent of digestion than with the unfractionated *C. bescii* exoproteome. Furthermore, we could recapitulate the activity of the exoproteome by utilizing a combination of only *Cb*Cel9A/Cel48A and two of the other enzymes such as *Cb*Cel9B/Man5A and *Cb*Xyn10A/Cel48B or *Cb*Cel9B/Man5A and *Cb*Man5B/Cel44A. Given the lack of mannan in the APCS substrate used one might expect to see even larger improvements in digestion on mannan rich substrates such as softwoods which are likely to be present in caldiphilic hot springs. The high extent of glucan and xylan conversion achieved by only three or four gene products suggests a genetically tractable enzyme cassette for conferring cellulolytic ability to high titer CBP microorganisms and for generally reducing the complexity of enzyme preparations in industry.

## Methods

### Strains, media, and culture conditions

*Caldicellulosiruptor bescii* strains and plasmids used in this study to express four kinds of multi-domain cellulases (Cbes_1867, Cbes_1857, Cbes_1859, and Cbes_1865) are listed in Additional file 1: Table S1. All *C. bescii* strains were grown anaerobically at 65 °C in liquid or on solid surface in low osmolarity defined (LOD) medium [26], with maltose or cellobiose (0.5% w/v) as the carbon source, final pH 6.8, for routine growth and transformation experiments [27]. For growth of uracil auxotrophs, LOD medium supplemented with 40 µM uracil was used. Liquid cultures were grown at 65 °C in anaerobic serum bottles having undergone iterative degassing: gassing cycles with nitrogen or argon. *E. coli* strain DH5α was used for plasmid DNA construction and preparation using standard techniques as described [28]. *E. coli* cells were cultured in LB broth supplemented with apramycin (50 μg/mL) and plasmid DNA was isolated using a Qiagen Mini-prep kit (Hilden, Germany). Total DNA from *Caldicellulosiruptor* strains was extracted using the Quick-gDNA™ MiniPrep (Zymo) as previously described [23].

### Construction of cellulases expression vectors and transformation into *C. bescii*

Plasmids in this study were generated using Q5 High-Fidelity DNA polymerase (New England BioLabs), *Bam*HI and *Sph*I restriction enzymes (New England BioLabs), and Fast-link™ DNA Ligase (Epicentre Technologies) according to the manufacturer’s instructions. Plasmid pJYW011 (Fig. 1b; Additional file 1: Table S1) was constructed by inserting the Cbes_1857 (1478 amino acid; GH10-CBM3_b_-CBM3_b_-GH48) open reading frame into pDCW170 [23], which contains the regulatory region of Cbes_2303 and a Rho-independent transcription terminator. The 7.975 kb DNA fragment was amplified with primers DC464 containing a *Bam*HI site and DC466 containing a *Sph*I site using pDCW170 as a template. A 4.45 kb DNA fragment containing the coding sequence of Cbes_1857 was amplified with JY020 containing a *Bam*HI site and JY021 containing a *Sph*I site using *C. bescii* chromosomal DNA as template DNA. These two linear DNA fragments were then digested with *Bam*HI and *Sph*I and ligated to construct pJYW011 (12.41 kb). Plasmid pJYW012 (Fig. 1c; Additional file 1: Table S1) is identical to pJYW011 except that it contains the entire coding sequence of Cbes_1859 (1294 amino acid; GH5-CBM3_b_-CBM3_b_-GH44). To make this change, a 3.894 kb DNA fragment containing the coding sequence of Cbes_1859 was amplified by PCR using primers JY022 (with *Bam*HI site) and JY023 (with *Sph*I site) using *C. bescii* genomic DNA as template. This fragment ligated with the 7.975 kb DNA fragment, which was used for the construction of pJYW011, to create pJYW012 (11.86 kb). Plasmid pJYW013 (Fig. 1d; Additional file 1: Table S1) was constructed by inserting the Cbes_1865 (1367 amino acid; GH9-CBM3_c_-CBM3_b_-CBM3_b_-GH5) open reading frame into pDCW173 [23], which contains the regulatory region of Cbes_2303, 6X Histidine tag, and a Rho-independent transcription terminator. The 7.969 kb DNA fragment was amplified with primers JY026 and JY027 using pDCW173 as a template. A 4.107 kb DNA fragment containing the coding sequence of Cbes_1865 was amplified with JY024 and JY025 using *C. bescii* chromosomal DNA as template DNA. These two linear DNA fragments were ligated via blunt-end ligation to construct pJYW013 (12.01 kb). Primers used for plasmid construction and confirmation are listed in Additional file 1: Table S2. *E. coli* strain DH5a cells were transformed by electroporation in a 2-mm-gap cuvette at 2.5 V and transformants were selected for apramycin resistance. The sequences of all plasmids were confirmed by automatic sequencing (Genewiz, NJ USA). All plasmids are available upon request.

To construct *C. bescii* expression strains JWCB057, JWCB058, and JWCB088, plasmids pJYW011, pJYW012, and pJYW013 were electrotransformed into JWCB029 (*ΔpyrFAΔldh::ISCbe4 Δcbe1ΔcelA*) cells as previously described [27]. Cultures, electro-pulsed with plasmid DNA (0.5–1.0 µg), were recovered in low osmolarity complex (LOC) growth medium [26] at 65 °C. Recovery cultures were transferred to liquid LOD medium without uracil to allow selection of uracil prototrophs. Cultures were plated on solid LOD media to obtain isolated colonies, and total DNA was isolated from transformants. PCR amplification using primers (DCB228 and DCB569) outside the gene cassette on the plasmid was used to confirm the presence of the plasmid with the gene of interest intact.

### Protein purification

*Cb*Cel9A/Cel48A, *Cb*Cel9B/Man5A, *Cb*Xyn10A/Cel48B, and *Cb*Man5B/Cel44A were expressed in *C. bescii* following the protocol reported earlier [11]. They were purified out of the *C. bescii* exoproteome using a 5 mL HisTrap fast flow column (GE) and were further purified using a Superdex 26/60 200 PG column in a 20 mM acetate pH 5.5 buffer containing 100 mM NaCl and 5 mM CaCl_2_ similarly to the protocol found in Brunecky et al. [11]. The *C. bescii* exoproteome was prepared following the purification protocol found in Yarbrough et al. [25]. *T. maritima* β-d-glucosidase was purchased from Megazyme (Bray, Ireland) and desalted using a Hi-trap 26/10 (GE life sciences) desalting column to remove ammonium sulfate stabilizer in a 20 mM acetate pH 5.5 buffer. Throughout the study, the total protein concentrations were measured by the Bradford method [29].

### Effect of pH and temperature on the activity of *Cb*Cel9A/Cel48A, *Cb*Cel9B/Man5A, *Cb*Xyn10A/Cel48B, and *Cb*Man5B/Cel44A

Enzyme activities were measured through sugar release quantification on 10 g/L (1% w/v) alkaline peroxide pretreated corn stover (APCS), the compositional analysis of this substrate can be found in Ref. [11]. *C. bescii* exoproteome and *Cb*Cel9A/Cel48A, were loaded at 1 mg/g glucan and *Cb*Xyn10A/Cel48B, *Cb*Cel9B/Man5A, and *Cb*Man5B/Cel44A were loaded at 5 mg/g glucan to carry out hydrolysis overnight at various temperatures and pH to determine the optimum conditions for enzyme function. The reaction was stopped by centrifugation of the reaction tube, separation of the supernatant containing soluble sugars, addition of 3,5-dinitrosalicylic acid (DNS) at a ratio of 2:1, and immediately followed by a 5 min boiling step. The solution was assayed at 540 nm to measure the release of reducing sugars from APCS using glucose as a standard [30].

The optimum temperature of each single enzyme and the *C. bescii* exoproteome was determined by incubation of the enzyme/APCS solution in 20 mM SEC buffer (pH 5.5) at different temperatures ranging from 70 to 90°C.

The optimum pH was determined using identical enzyme and substrate loadings but at a constant temperature of 75 °C. Reactions were conducted in 20 mM SEC buffers ranging from pH 4.0 to pH 7.0.

### *p*NP and AZCL assay

To rapidly screen a variety of potential activities we assayed the enzymes against both 4-nitrophenol (*p*NP) and AZCL dyed substrates. The substrates selected were 4-nitrophenyl-b-d-xylopyranoside (*p*NP-X), 4-nitrophenyl-β-d-cellobioside (*p*NP-C), 4-nitrophenyl β-d-glucopyranoside (*p*NP-G), 4-nitrophenyl-β-d-mannopyranoside (*p*NP-Man), 4-nitrophenyl-β-d-galactopyranoside (*p*NP-Gal), 4-nitrophenyl-β-d-maltopyranoside (*p*NP-mal), 4-nitrophenyl-alpha-l-arabinofuranoside (*p*NP-arab), and 4-nitrophenyl-α-l-rhamnopyranoside (*p*NP-rham). AZCL substrates tested include: AZCL-xyloglucan, AZCL-curdlan, AZCL-potato galactan, AZCL-pachyman, AZCL-amylose, AZCL-dextran, AZCL-B-glucan, AZCL wheat arabinoxlyan, AZCL-galactomannan, and AZCL Oatspelt Xylan. PNP assays were performed in 96 well plates at 75 °C, in 50 mM pH 5.5 acetate buffer with 5 mM CaCl_2_ added, for 30 min with the noted enzymes at a concentration of 0.5 mg/mL, then quenched and absorbance recorded at 405 nm using a Spectromax plate reader. AZCL assays were performed in 96 well plates at 75 °C, in 50 mM pH 5.5 acetate buffer with 5 mM CaCl_2_ added, for 1 h at a concentration of 0.25 mg/mL. Activity was noted by visual observation of dye release.

### Individual enzyme product detection

Single enzymes were used on alkaline peroxide pretreated corn stover (APCS) at 10 mg/g glucan for 24 h at 75 °C. Analysis of samples for cello and xylooligomers was performed on an Acquity Ultra Performance Liquid Chromatography (UPLC) system (Waters Co., Milford, MA) equipped with a TQD Mass Spectrometer (MS) and Evaporative Light Scattering Detector (ELSD). Waters Masslynx 4.1 software version was used to collect and investigate the analytes of interest. The samples were injected at 10 µL per run, separated and detected using an UPLC system equipped with a TQD MS and ELSD detector. The chromatography system utilized a Shodex Sugar SZ5532 (Zinc) column (Showa Denko K.K., Japan) at 6 × 150 (mm), 6 µm particle size, to sufficiently separate the oligomers of interest. The column temperature was maintained at 60 °C, the buffers used to separate the analytes was 0.1% formic acid in water (A)/0.1% formic acid in acetonitrile (B) while keeping samples at 4 °C within the Acquity Autosampler. The separation was carried out using a gradient program of: (A) = 20% and (B) = 80% at time *t* = 0; (A) = 17% and (B) = 83% at *t* = 9 min; (A) = 30% and (B) = 70% at *t* = 25 min; (A) = 40% and (B) = 60% at *t* = 40 min; (A) = 20% and (B) = 80% at *t* = 45 min. The flow rate was held constant at 0.9 mL/min and each sample was split approximately around 3:1 between the MS and ELSD detectors. The ELSD parameters were set as follows: drift tube temperature was set to 76 °C and the gain to 10. The gas pressure was set to 28 psi, while the nebulizer mode, heater–cooler, and data channel were disabled. The TQD MS system was setup with electrospray under negative mode with a cone voltage of 30 V, capillary voltage at 3000 V, desolvation temperature at 300 °C and a source temperature of 110 °C. The MS was calibrated and tuned with Sodium Iodide and subsequent samples where scanned from 50 to 2000 amu with a 1 s scan time.

### Biomass solubilization assay

The alkaline pretreated corn stover (APCS) was from the same batch as reported in Brunecky and co-workers [11]. For the mono-component biomass digestions we utilized a loading of 15 mg/g glucan loading for all enzymes tested supplemented with beta glucosidase from *T. maritima* (Megazyme) at a loading of 0.5 mg/g glucan.

The enzyme loadings in binary, ternary, and quaternary mixtures used to mimic the ratios of these CAZymes in the exoproteome as analyzed by proteomic analysis [13] can be found in Additional file 1: Tables S4, S5. The *C. bescii* exoproteome used for this digestion experiment was loaded at 15 mg/g. All digests were supplemented with 0.5 mg/g glucan Beta glucosidase from *T. maritima* (Megazyme). CelA mix digestions were performed at 75 °C at pH 5.5. All digestions were conducted at a total initial solids loading of 1%.

Digestions were run continuously for 5 days with sampling at various time points. Enzymes were inactivated by boiling for 15 min after which samples were filtered through 0.45 μm Acrodisc syringe filters. The released sugars were analyzed by HPLC. Samples were injected at 20 μL volume and run on an Agilent 1100 HPLC system equipped with a BioRad Aminex HPX-87H 300 mm × 7.8 mm column heated to 55 °C. A constant flow of 0.6 mL/min was used with 0.1 N H_2_SO_4_ in water as the mobile phase to give optimal sugar separation. Glucose, xylose, cellobiose and xylobiose were quantified against independent standard curves and converted to anhydrous glucan equivalent and the results are reported as anhydrous glucan converted. All experiments were performed in triplicate and the resulting extents of conversion are shown as percent glucan or xylan converted.

## Additional file

**Additional file 1.** Additional tables and figures.
