# Supplementary material for: High activity CAZyme cassette for improving biomass degradation in thermophiles
Source: Biotechnol Biofuels. 2018 Feb 1;11:22. doi: 10.1186/s13068-018-1014-2 (PMC5793385; doi:10.1186/s13068-018-1014-2)
Supplement: Supplementary file 1 — Additional file 1. Additional tables and figures. [file 13068_2018_1014_MOESM1_ESM.docx]

| **Table S1.** *Caldicellulosiruptor bescii* strains and plasmids used in this study | | | |
| --- | --- | --- | --- |
| **Strains** | | **Description** | **Source** |
| JWCB029 | *ΔpyrFAΔldh::ISCbe4 Δcbe1 ΔcelA* (ura^-^/5-FOA^R^) | | ([*1*](#_ENREF_1)) |
| JWCB047 | JWCB029 harboring pDCYB173 | | ([*1*](#_ENREF_1)) |
| JWCB057 | JWCB029 harboring pJYW011 | | This study |
| JWCB058 | JWCB029 harboring pJYW012 | | This study |
| JWCB088 | JWCB029 harboring pJYW013 | | This study |
|  | |  |  |
| **Plasmids** | |  |  |
| pDCW170 | *C. bescii* expression vector for Cbes_1867 (Apramycin^R^) | | ([*1*](#_ENREF_1)) |
| pDCW173 | *C. bescii* expression vector for Cbes_1867 (Apramycin^R^) | | ([*1*](#_ENREF_1)) |
| pJYW011 | *C. bescii* expression vector for Cbes_1857 (Apramycin^R^) | | This study |
| pJYW012 | *C. bescii* expression vector for Cbes_1859 (Apramycin^R^) | | This study |
| pJYW013 | *C. bescii* expression vector for Cbes_1865 (Apramycin^R^) | | This study |

**Table S2.** List of primers used in this study. The underlined sequences indicate the recognition sites of the corresponding restriction enzymes.

| Name | Sequence (5’ → 3’) | Restriction  enzyme | Description |
| --- | --- | --- | --- |
| DC228 | ATCATCCCCTTTTGCTGATG | - | To confirm transformant |
| DC569 | AGAGTAGAGCGTGATGACATAGA | - | To confirm transformant |
| DC464 | GGATCCCTCACCAAACCTCCTTGTATGAT | BamHI | To construct pJYW011/012 |
| DC466 | GCATGCCATCACCATCACCATCACTAATAATAAAGCTGAAATAAAAGAGGGTGAGA | SphI | To construct pJYW011/012 |
| JY020 | GGATCCATGATGAAGAAATTAGTTAAAATTATAACTCACG | BamHI | To construct pJYW011 |
| JY021 | GCATGCTTGATTGCCAAACAGTATTTCATATGTTGC | SphI | To construct pJYW011 |
| JY022 | GGATCCATGAGAGTAAAAACAAAAATGGGGAAG | BamHI | To construct pJYW012 |
| JY023 | GCATGCTTTAGTTTGTACTGAGGTTGAATATAAAACG | SphI | To construct pJYW012 |
| JY024 | ATGCTTAAACTAAAAAGAGCAATAAAAATGATTA | - | To construct pJYW013 |
| JY025 | TTCAGCACCAATCGCATTAGTTTTATACCA | - | To construct pJYW013 |
| JY026 | CATCACCATCACCATCACTAATAATAAAG | - | To construct pJYW013 |
| JY027 | ATCATACAAGGAGGTTTGGTGAGGGATCC | - | To construct pJYW013 |

**Table S3:** Product profiles of individual component enzymes: Presence of each xylo-oligomer product released by each enzyme after a 24hr hydrolysis of APCS, without the presence of Beta-Glucosidase. No products past DP8 were detected for these enzymes.

| **Species** | **(DP)** | ***Cb*Cel9A/Cel48A** | ***Cb*Xyn10A/Cel48B** | ***Cb*Cel9B/Man5A** | ***Cb*Man5B/Cel44A** |
| --- | --- | --- | --- | --- | --- |
| Xylobiose | DP2 | x | x | x | x |
| Xylotriose | DP3 | x | x | x | x |
| Xylotetraose | DP4 | x | x | x | x |
| Xylopentaose | DP5 | x | x | x | x |
| Xylohexaose | DP6 | x | x | x | x |
| Xyloheptaose | DP7 | ND | x | x | x |

**Table S4:** Enzyme loadings for the digestion experiments reported in Figure 5c,d

|  | Figures 5c,d | | | |
| --- | --- | --- | --- | --- |
|  | *Cb*Cel9A/Cel48A | *Cb*Cel9A/Cel48A  *+ Cb*Cel9B/Man5A | *Cb*Cel9A/Cel48A +  *Cb*Xyn10A/Cel48B | *Cb*Cel9A/Cel48A +  *Cb*Man5B/Cel44A |
| *Cb*Cel9A/Cel48A | 15mg/g | 11.5mg/g | 11.2mg/g | 13.5mg/g |
| *Cb*Cel9B/Man5A | ____ | 3.5mg/g | ____ | ____ |
| *Cb*Xyn10A/Cel48B | ____ | ____ | 3.8mg/g | ____ |
| *Cb*Man5B/Cel44A | ____ | ____ | ____ | 1.5mg/g |

**Table S5:** Enzyme loadings for the digestion experiments reported in Figure 5c,d

|  | Figures 6e ,f | | | |  |
| --- | --- | --- | --- | --- | --- |
|  | *Cb*Cel9A/Cel48A | Cassette 1 | Cassette 2 | Cassette 3 | Cassette 4 |
| *Cb*Cel9A/Cel48A | 15mg/g | 9.4mg/g | 10.6mg/g | 10.7 mg/g | 8.8 mg/g |
| *Cb*Cel9B/Man5A | ____ | 2.8mg/g | 3.2mg/g | ____ | 2.6 mg/g |
| *Cb*Xyn10A/Cel48B | ____ | 2.8mg/g | ____ | 3.1 mg/g | 2.6 mg/g |
| *Cb*Man5B/Cel44A | ____ | ____ | 1.2mg/g | 1.2 mg/g | 1.0 mg/g |

**
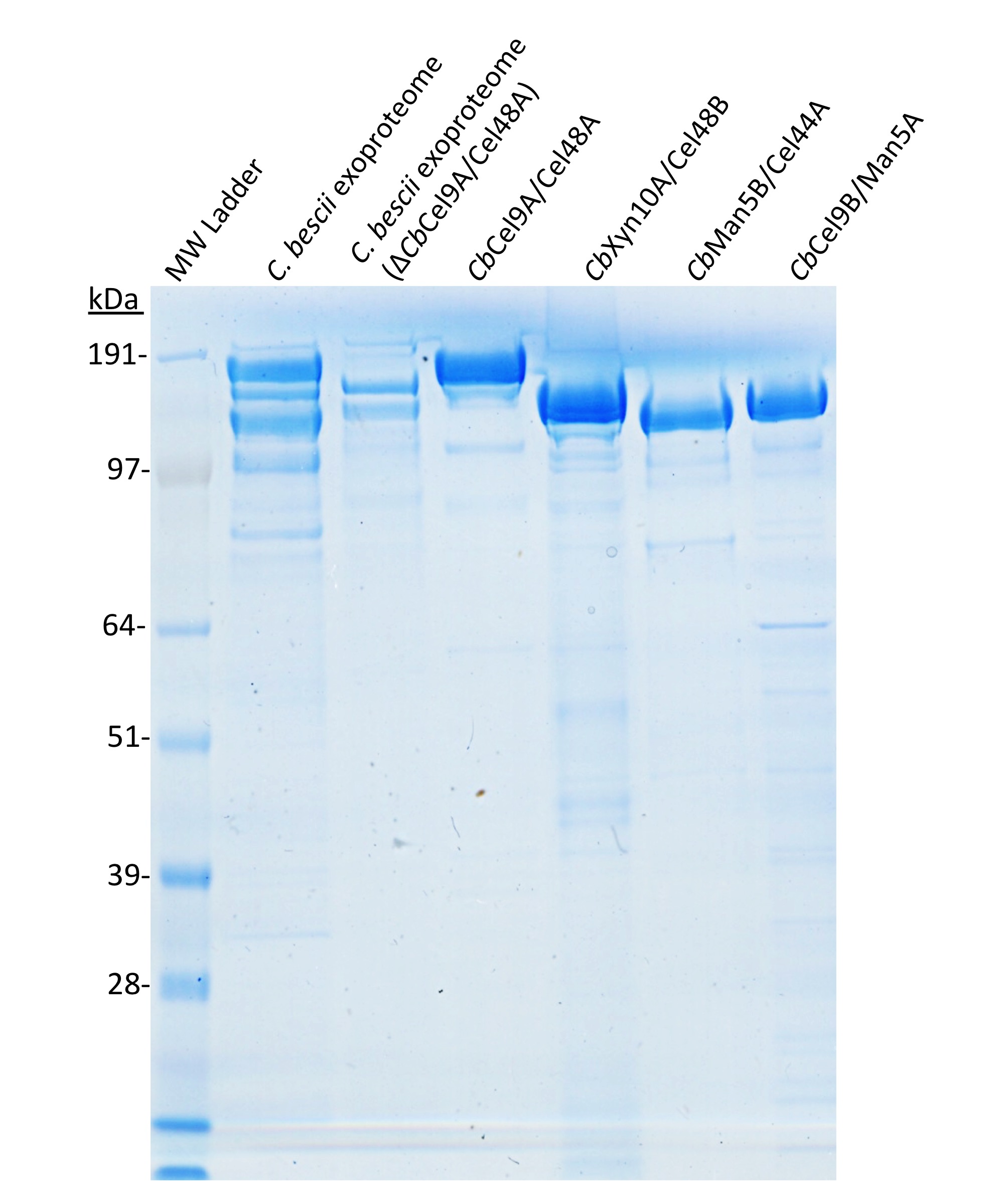
**

**Figure S1:** Sodium dodecyl sulfate (SDS) gel of the *C.bescii* exoproteome, *C.bescii* exoproteome with *Cb*Cel9A/Cel48A deleted (from reference ([*2*](#_ENREF_2))), *Cb*Cel9A/Cel48A, *Cb*Xyn10A/Cel48B, *Cb*Man5B/Cel44A, and *Cb*Cel9B/Man5A.


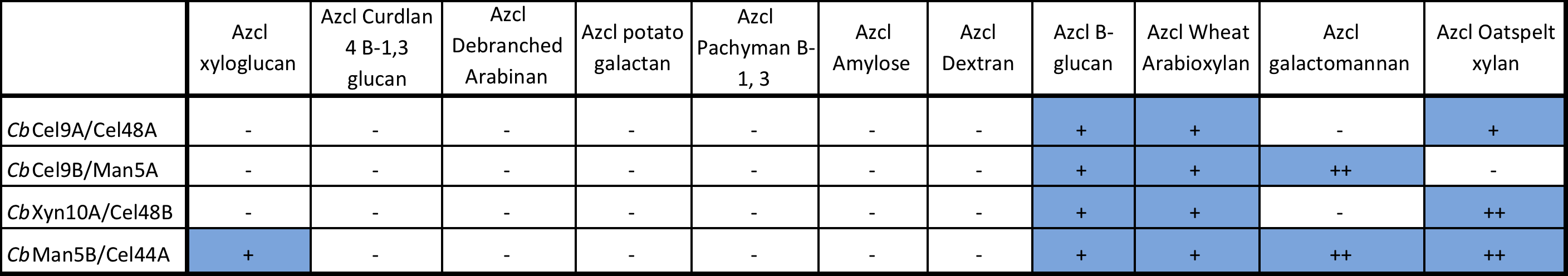


**Figure S2:** Table of observed AZCL dye release assay on a variety of dyed-substrates


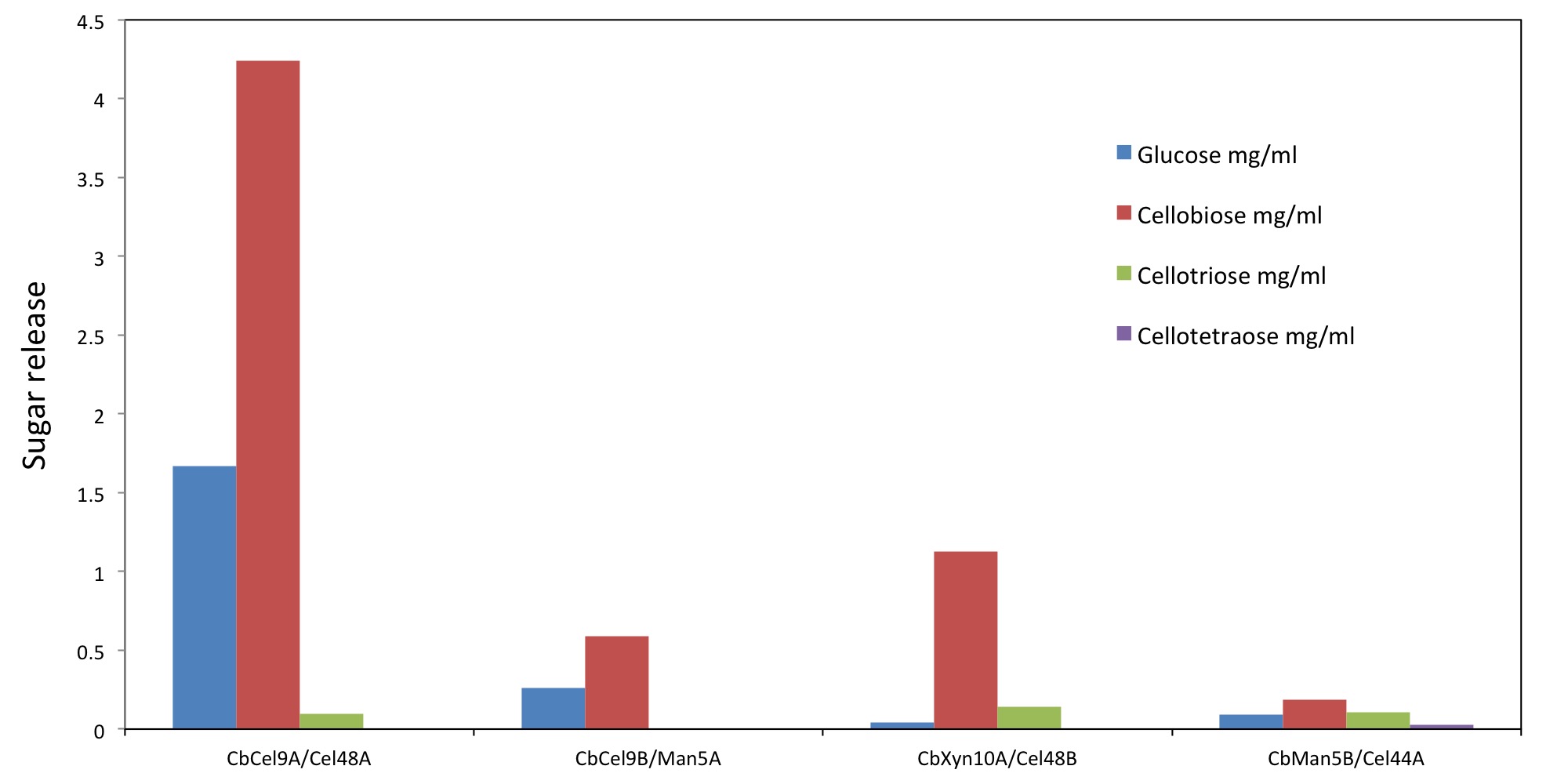


**Figure S3**: Product profiles of individual component enzymes: Concentration of each cello-oligomer product in mg/ml released by each enzyme after a 24hr hydrolysis of APCS, without the presence of Beta-Glucosidase.


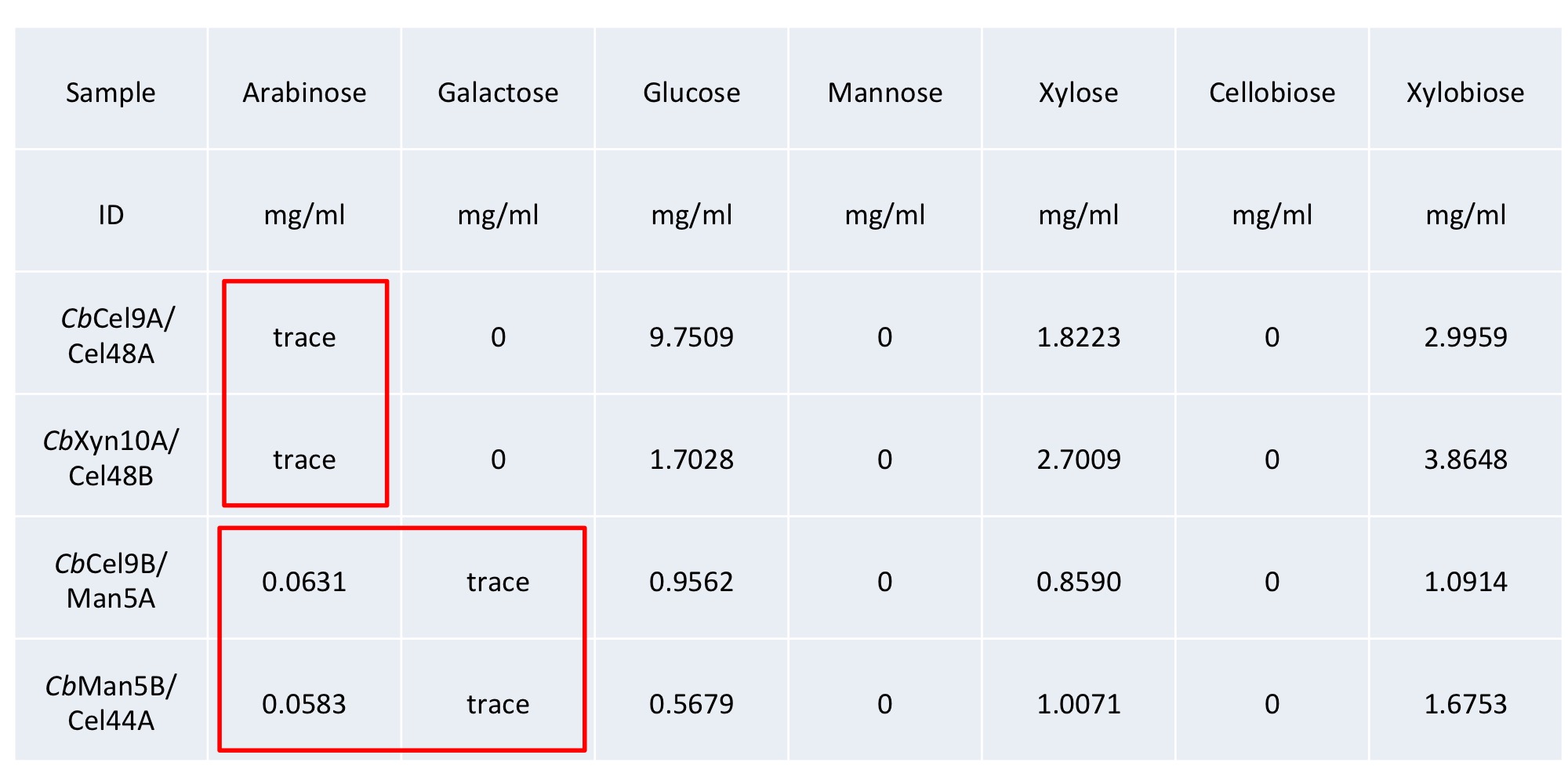


**Figure S4.** Anion exchange chromatography with pulsed amperometric detection analysis of final time points of the 5 day single enzyme digestions shown in figure 4a illustrating small release of arabinose and galactose.

1. D. Chung *et al.*, Homologous expression of the Caldicellulosiruptor bescii CelA reveals that the extracellular protein is glycosylated. *PLoS One* **10**, e0119508 (2015).

2. J. Young, D. Chung, Y. J. Bomble, M. E. Himmel, J. Westpheling, Deletion of Caldicellulosiruptor bescii CelA reveals its crucial role in the deconstruction of lignocellulosic biomass. *Biotechnology for biofuels* **7**, 142 (2014).
